# Posterior segment eye disease in sub-Saharan Africa: review of recent population-based studies

**DOI:** 10.1111/tmi.12276

**Published:** 2014-01-31

**Authors:** Andrew Bastawrous, Philip I Burgess, Abdull M Mahdi, Fatima Kyari, Matthew J Burton, Hannah Kuper

**Affiliations:** 1 International Centre for Eye Health, London School of Hygiene & Tropical MedicineLondon, UK; 2 Malawi-Liverpool-Wellcome Trust Clinical Research Programme, Queen Elizabeth Central HospitalBlantyre, Malawi; 3 Department of Ophthalmology, Abubakar Tafawa Balewa University Teaching HospitalBauchi, Nigeria; 4 Department of Ophthalmology, College of Health Sciences, University of AbujaAbuja, Nigeria; 5 Moorfields Eye HospitalLondon, UK; 6 International Centre for Evidence in Disability, London School of Hygiene & Tropical MedicineLondon, UK

**Keywords:** glaucoma, diabetic retinopathy, age-related macular degeneration, posterior segment eye disease, prevalence, incidence, blindness, visual impairment, Africa

## Abstract

**Objective:**

To assess the burden of posterior segment eye diseases (PSEDs) in sub-Saharan Africa (SSA).

**Methods:**

We reviewed published population-based data from SSA and other relevant populations on the leading PSED, specifically glaucoma, diabetic retinopathy and age-related macular degeneration, as causes of blindness and visual impairment in adults. Data were extracted from population-based studies conducted in SSA and elsewhere where relevant.

**Results:**

PSEDs, when grouped or as individual diseases, are a major contributor to blindness and visual impairment in SSA. PSED, grouped together, was usually the second leading cause of blindness after cataract, ranging as a proportion of blindness from 13 to 37%.

**Conclusions:**

PSEDs are likely to grow in importance as causes of visual impairment and blindness in SSA in the coming years as populations grow, age and become more urban in lifestyle. African-based cohort studies are required to help estimate present and future needs and plan services to prevent avoidable blindness.

## Introduction

### Non-communicable diseases in low- and middle-income countries

In recent decades, there has been a marked rise in life expectancy that has contributed to a major epidemiological shift in populations worldwide (Lopez *et al*. 2006). These changes will increasingly lead to major public health issues in low- and middle-income countries (LMIC; Mathers & Loncar 2006). Current projections suggest that non-communicable diseases (NCDs) will contribute to two-thirds of global mortality by the year 2030 (Mathers & Loncar 2006). NCDs in LMIC have shown substantial variation in prevalence, incidence, natural history and risk factors compared with NCDs in populations in high-income countries (Boutayeb 2006).

### Visual impairment and blindness

285 million people are visually impaired (VI) worldwide, (severe visual impairment (SVI) defined as presenting visual acuity (PVA) <6/60 but ≥3/60, moderate VI defined as PVA <6/18 but ≥6/60) of whom 39 million are blind (presenting visual acuity <3/60 in the better eye; Pascolini & Mariotti 2012). Approximately 90% of those worldwide with VI live in low-income countries. NCDs are the leading causes of VI, in part due to the successful control of infectious diseases. VI is ranked sixth in the top ten causes of burden of disease in terms of disability-adjusted life-years (DALYs) in low-income, middle-income and high-income countries (Chiang *et al*. 2006). The sum of DALYs from VI is 66 290 000 (4.3% of total), just below HIV/AIDS at 71 460 000 (4.7%).

The number of people visually impaired in the World Health Organization (WHO) African region is estimated to be 26 million, of whom almost 6 million are blind. This is based on estimates from population-based studies in Botswana, Cameroon, Eritrea, Ethiopia, Gambia, Ghana, Kenya, Mali, Nigeria, Rwanda, Uganda and Tanzania (Pascolini & Mariotti 2012). Despite Africa having one of the highest prevalences of blindness, it is the most underserved continent in terms of human resources available to treat and manage eye disease (Resnikoff *et al*. 2012), with the greatest gap between existing need and provision (Bastawrous & Hennig 2012).

**Figure 1:**
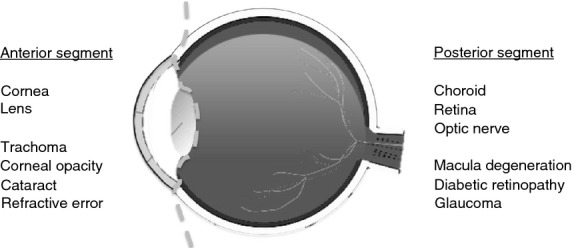
Cross-sectional diagram of the eye demonstrating the anterior and posterior segments and their potential diseases.

In 2010, the WHO reported the leading causes of visual impairment (VI) and blindness (Pascolini & Mariotti 2012). Of these, three of the nine listed leading causes are NCDs which are posterior segment in location, (i.e. affecting the back of the eye). Posterior segment eye disease (PSED) epidemiologically is commonly defined as diseases of the retina, choroid and optic nerve and primarily includes: glaucoma, age-related macular degeneration (AMD) and diabetic retinopathy (DR). These three conditions are the focus of this paper but do not constitute all PSEDs. See Figure 1.

### PSED and VISION2020

VISION2020 is the global initiative for the elimination of avoidable blindness, launched in 1999, jointly by WHO and the International Agency for the Prevention of Blindness (IAPB) and provides technical support and advocacy to prevention of blindness activities worldwide. It aims over two decades to prevent 100 million people from becoming blind.

VISION2020 has largely focused on the elimination of anterior segment diseases, primarily cataract, as it alone causes almost half of blindness and is amenable to cure through surgery. VISION2020 has not focused on PSED to date mostly due to a lack of data on the magnitude of these conditions and lack of cost-effective treatment options. This review aims to establish the magnitude of visual impairment and blindness in SSA that can be attributed to PSED.

## Materials and methods

Our literature search was conducted for the years 1966 to September 2012 using PubMed. Keywords used included the following: posterior segment eye disease, glaucoma, age-related macular degeneration, diabetic retinopathy, correctable visual impairment, preventable, avoidable, Africa (MeSH), aphakia, blindness, visual impairment, prevalence and population.

Studies were selected for inclusion if they were population based, performed in sub-Saharan Africa with a sample size >1000, reported visual acuity impairment with its causes, had a high participation rate (>80% of the targeted sample) and presented results using the standard WHO categories of VA. WHO definitions of visual impairment are used (WHO/ICD-10 2007). We also searched reference lists of studies meeting inclusion criteria. Only published data were included.

All-cause prevalence (and 95% confidence interval [CI]) of blindness, SVI and moderate VI was extracted from each study, as well as the proportion of blindness, SVI and moderate VI due to PSED (grouped or as single diseases when available); then, the prevalence of blindness, SVI and moderate VI due to PSED was calculated from these estimates.

## Results

### Search results

In total, the initial search criteria identified 112 potential manuscripts for inclusion. Review of the abstracts reduced this to 39 potential studies, of which 17 surveys, from 13 SSA countries, encompassing 88 067 individuals were included for analysis having fully met the pre-specified search criteria. Data from the following countries are presented: Burundi (Kandeke *et al*. 2012), Cameroon (Oye *et al*. 2006; Oye & Kuper 2007), Eritrea (Muller *et al*. 2011), Ethiopia (Berhane *et al*. 2007), Ghana (Budenz *et al*. 2012), Guinea (Moser *et al*. 2002), Kenya (Mathenge *et al*. 2007a, 2012), Malawi (Kalua *et al*. 2011), Nigeria (Adegbehingbe *et al*. 2006; Abdull *et al*. 2009), Rwanda (Mathenge *et al*. 2007b), South Sudan (Ngondi *et al*. 2006), Tanzania (Kikira 2007; Habiyakire *et al*. 2010) and Uganda (Mbulaiteye *et al*. 2002).

### Posterior segment eye disease

Although PSEDs are frequently collated in SSA-based epidemiological studies and presented as a single entity or group of conditions, they are clinically and pathophysiological distinct. The most common methodological approach deployed in SSA population-based studies, the rapid assessment of avoidable blindness (RAAB; Dineen *et al*. 2006), is not sufficiently sensitive to differentiate posterior segment causes of low vision and hence presented results are often collated.

### Posterior segment eye disease in Africa

Cross-sectional population-based studies from the last two decades performed in Africa have shown PSED to be consistently the second (and occasionally the most common) leading cause of blindness. This includes studies from Kenya (Mathenge *et al*. 2007a, 2012), Nigeria (Rabiu & Muhammed 2008; Abdull *et al*. 2009), Tanzania (Kikira 2007; Habiyakire *et al*. 2010), Rwanda (Mathenge *et al*. 2007b), Cameroon (Oye *et al*. 2006; Oye & Kuper 2007), Ghana (Guzek *et al*. 2005), Guinea (Moser *et al*. 2002), Burundi (Kandeke *et al*. 2012) and Ghana (Budenz *et al*. 2012; See Table 1). No longitudinal data on PSED from population-based studies in Africa have been published. A single cohort in Uganda has 3-year cumulative incidence data on visual impairment, (age-standardised incidence rate of 13.2, per 1000 PY) with AMD and glaucoma amongst the leading causes of visual loss in new cases (Mbulaiteye *et al*. 2003). However, no baseline clinical phenotyping data were collected in eyes initially without visual impairment, so early asymptomatic disease was not excluded.

**Table 1:** Reviewed studies

Country	Level	Year published	Sample size *(number examined)*	Response rate (%)	Age (years)	Primary cause of blindness	Secondary cause of blindness	Equipment used for diagnosis	References
Burundi	Provincial	2012	3684	97	≥50	Cataract (55%)	PSED (37%)	Not stated	Kandeke *et al*. (2012)
Cameroon	Limbe	2007	2215	92.3	≥40	Cataract (62%)	PSED (25%)	Direct ophthalmoscope	(Oye and Kuper (2007)
Cameroon	Muyuka	2006	1787	89.3	≥40	PSED (29%)	Cataract (21%)	Direct ophthalmoscope	Oye *et al*. (2006)
Eritrea	National	2011	3163	95.9	≥50	Cataract (55%)	Glaucoma (15%)	Portable slit lamp	Muller *et al*. (2011)
Ethiopia	National	2007	25650	85.4	All	Cataract (50%)	Trachoma (8%)	Direct ophthalmoscope	Berhane *et al*. (2007)
Ghana	City	2012	5603	82.3	≥40	Cataract (44%)	Glaucoma (22%)	Slit lamp/fundus camera	Budenz *et al*. (2012)
Guinea	District, Bioko	2002	3218	NS	All	Cataract (61%)	Macular Affection (21%)	Slit lamp	Moser *et al*. (2002)
Kenya	District, Nakuru (RAAB)	2007	3503	92.6	≥50	Cataract (42%)	PSED (30%)	Direct ophthalmoscope	Mathenge *et al*. (2007a)
Kenya	District, Nakuru	2012	4414	88.1	≥50	Cataract (45%)	PSED (32%)	Slit lamp/fundus camera	Mathenge *et al*. (2012)
Malawi	District	2011	3430	95.7	≥50	Cataract (48%)	Glaucoma (16%)	Direct ophthalmoscope	Kalua *et al*. (2011)
Nigeria	National	2009	13599	89.9	≥40	Cataract (43%)	Glaucoma (17%)	Slit lamp/fundus camera	Abdull *et al*. (2009)
Nigeria	Local Government Area	2008	2424	93.6%	≥50	Cataract (46%)	Surgical complications (20%)	Direct ophthalmoscope	Rabiu (2008)
Rwanda	Western province	2007	2206	98	≥50	Cataract (65%)	PSED (20%)	Direct ophthalmoscope	Mathenge *et al*. (2007b)
South Sudan	District	2006	2499	84.6	≥5	Cataract (41%)	Trachoma (35%)	Torch	Ngondi *et al*. (2006)
Tanzania (Kilimanjaro)	Regional	2010	3436	95.5	≥50	Cataract (51%)	PSED (36%)	Direct ophthalmoscope	Habiyakire *et al*. (2010)
Tanzania (Zanzibar)	Island	2007	3160	98.8	≥50	Cataract (67%)	PSED (25%)	Direct ophthalmoscope	Kikira (2007)
Uganda	15 neighbouring villages	2002	4076	98.9	≥13	Glaucoma (39%)	Cataract (23%)	Direct ophthalmoscope	Mbulaiteye *et al*. (2002)

NS, Not stated. RAAB, rapid assessment of avoidable blindness, PSED, posterior segment eye disease.

All studies were cross-sectional, population-based studies, which used cluster random sampling.

The majority of available prevalence data in Africa come from the rapid assessment of avoidable blindness (RAAB) methodology (Dineen *et al*. 2006). Although RAAB is a validated survey method (Mathenge *et al*. 2012), it has a limitation in common with more comprehensive surveys such as the Nigeria study (Dineen *et al*. 2008) in that detailed eye examinations are only performed in those found to have impairment of their visual acuity. As glaucoma patients usually lose central vision at the end stage of the disease, they are frequently missed unless visual field assessment is performed. Furthermore, ophthalmic assessment in RAAB relies on direct ophthalmoscopy, constraining diagnostic accuracy, so that the diseases are pragmatically grouped together as one unit.

The majority of these surveys have used the WHO coding instructions, which use the ‘principal disorder responsible for visual loss in the individual after considering disorders in either eye which are most amenable to treatment or prevention’ (World Health Organization 1988). In other words, if a patient has PSED coexistent with cataract, it will be deemed that cataract is the primary cause of blindness/VI. Therefore, most VI prevalence data available in which cataract or refractive error is the primary cause will underestimate the prevalence of PSED at all levels of visual acuity.

### Glaucoma in Africa

#### Prevalence

Current estimates suggest that there are 6.5 million people with glaucoma at all levels of vision in sub-Saharan Africa (SSA) with a projected increase to 8.4 million in 2020 (Quigley & Broman 2006). Glaucoma is estimated to be the second leading cause of blindness in Africa (Cook 2009). These estimates undertaken by Quigley and Broman (2006) are based on seven population-based studies, of which two examined individuals of African descent living outside of the African continent: in Baltimore, USA (Leske *et al*. 1994) and Barbados (Tielsch *et al*. 1991a) which has multiple limitations for inferring data. Of the five based in Africa, three were undertaken in South Africa (Salmon & Martell 1994; Rotchford & Johnson 2002; Rotchford *et al*. 2003), one in Ghana (Ntim-Amponsah *et al*. 2004) and one in Tanzania (Buhrmann *et al*. 2000). The studies used varying sampling methods and criteria for diagnosis of glaucoma.

No specific and sensitive test for glaucoma exists. Current reference standard diagnosis requires expensive visual field-testing equipment with expert interpretation of the optic disc and visual field findings. Standardised definitions and classifications of glaucoma in recent years have allowed for better prevalence estimates and comparisons between populations (Foster *et al*. 2002).

Glaucoma may be congenital or acquired and further subclassified into open-angle and closed-angle based on the mechanism by which aqueous outflow from the eye is compromised. The ‘angle’ refers to the junction between cornea and iris, which forms an angle of varying degree in each eye. Generally speaking, in glaucoma, when this angle is large and the structures within it are visible on clinical examination (gonioscopy), it is termed ‘open-angle glaucoma’ and when these structures are limited or not visible due to a narrow angle, it is termed ‘closed- or narrow-angle glaucoma’ (Kanski 2007). Primary open-angle glaucoma (POAG) disproportionately affects individuals of African descent (Quigley & Broman 2006)and is difficult to diagnose in early disease, and when diagnosis is confirmed, there is still debate on the best management in the context of limited resources and prospects for long-term follow-up (Quigley *et al*. 2000). Narrow-angle glaucoma prevalence is not well reported in African populations, this is in large part due to gonioscopy not being performed in the frequently used RAAB methodology and also in other more comprehensive surveys (Mathenge *et al*. 2012).

People of African descent (not living in Africa) have a higher prevalence of glaucoma, are more likely to develop glaucoma at an early age with more aggressive disease and have a higher risk of glaucoma related blindness than Caucasians or Asians (Mason *et al*. 1989; Tielsch *et al*. 1991b). It is therefore vital that the epidemiology of glaucoma is investigated in more detail in various populations in Africa.

Comprehensive reviews on glaucoma in Africa were published in 2009 (Cook 2009) and 2013 (Kyari *et al*. 2013), no new data from African population-based studies have since been published since 2009. The authors are aware of awaited data to be published from study groups in Nigeria (Dineen *et al*. 2008), Ghana and Kenya (Mathenge *et al*. 2012).

Current data on glaucoma underestimate the true prevalence, as many cases of glaucoma have preservation of central vision and do not include visual field assessment (Cook 2009). Furthermore, preferential coding of cataract due to its reversible nature often means that glaucoma is not assigned as the primary cause of blindness in a patient with visual loss from coexistent glaucoma and lens opacity, as per WHO criteria (World Health Organization 1988).

#### Incidence

It is assumed that incidence of glaucoma in Africa will most closely reflect that of the Barbados Eye Study, whose enrolled participants were of West African descent (Leske *et al*. 2001, 2007). All other studies with data on glaucoma incidence have been conducted in largely Caucasian populations: the Ponza Eye Study (Cedrone *et al*. 2012), the Dalby Eye Study (Bengtsson 1991), the Blue Mountain Eye Study (Chandrasekaran *et al*. 2006), the Melbourne Visual Impairment Study (Dimitrov *et al*. 2003) and the Rotterdam Eye Study (de Voogd *et al*. 2005). Annual incidence of new glaucoma in these studies varied from 0.1 to 0.6%, the highest being in the Barbados Eye Study which was largely made up of people of African descent. To date, no data on incident glaucoma or glaucoma progression from population-based studies in Africa are available.

### Diabetic retinopathy in Africa

#### Prevalence

Diabetes is a major threat to global public health. The estimated prevalence of diabetes worldwide was 285 million in 2010, representing 6.4% of the world's adult population, with a prediction that by 2030 there will be 438 million people with diabetes (DF 2009). The most substantial increases (7 to 15 million, 111%) are expected to be in Africa and the Middle East as a result of various factors including population growth, ageing, urbanisation, dietary changes and the increase in obesity and sedentary lifestyles in these regions (King & Herman 1998).

Although no data exist from population-based studies (PBS) in Africa directly comparing ethnic variation as a risk for DR, a hospital-based study in South Africa estimated the prevalence of DR amongst patients with adult-onset diabetes attending a large community hospital to be similar in patients of African (37%), European (41%) or Indian (37%) heritage. However, ‘severe DR’ (study specific classification) was significantly more frequent in Africans (52%) and Indians (41%) than Europeans (26%; Kalk *et al*. 1997). The predicted rise in proportion of adults suffering from diabetes will inevitably lead to an increase in the prevalence of DR (Williams *et al*. 2004).

The detection of DR in Africa remains a challenge in part due to a lack of necessary equipment and skilled manpower (Rotimi *et al*. 2003). The authors of this review [also cited in reference: (Burgess *et al*. 2013)] identified two high-quality, population-based, cross-sectional studies reporting DR prevalence in Africa (but not SSA). The Diabetes in Egypt project (1993; Herman *et al*. 1998) reported the proportion of DR and PDR in individuals with diabetes to be 31.6% and 0.9%, respectively. The Mauritius diabetes complication study (Dowse *et al*. 1998) reported 30.2% DR and 1.3% PDR; the prevalence of PDR in subjects with known diabetes was 2.3%. These figures are comparable with prevalence estimates reported in recent American and European studies.

Egypt and Mauritius are ethnically and demographically very different to most countries of sub-Saharan Africa; the findings of these studies should be generalised to other settings with caution.

There are also estimates of the prevalence of DR amongst diabetics from high-quality clinic-based studies in Africa. Very high prevalences of DR, PDR and maculopathy have been reported. A study from Malawi reported 32.0% DR, 5.7% PDR, 15% sight-threatening maculopathy (Glover *et al*. 2012). Two separate studies from South Africa have found comparable results: Mash *et al*. found 62.4% DR, 6.1% PDR and 15.2% with any maculopathy (Mash *et al*. 2007); Rotchford *et al*. found DR 40.3%, PDR 5.6%, 10.3% CSME (Rotchford 2002).

Evidence from unpublished data supports urbanisation as a risk factor for DR. Slit-lamp assessment of the retina assessing DR in a South-African PBS (Rotchford & Johnson 2002; Rotchford *et al*. 2003) demonstrated a 0.7% prevalence of DR (NPDR 0.6%, PDR 0.1%) in rural communities and a 2.1% prevalence of DR (NPDR 1.8%, PDR 0.3%) in urban communities (A. Rotchford, unpublished data).

Estimates of the proportion of African patients with diabetes who are visually impaired are high even compared with older European and American studies. The population-based Nigerian national blindness and visual impairment survey was conducted between 2005 and 2007 (Abdull *et al*. 2009). DR was identified as the primary cause of visual impairment in 0.29% of 3129 subjects with uncorrected VA worse than 6/12 and in 0.5% of those with acuity less than 3/60. This study is likely to underestimate the visual impact of DR as examiners were instructed to preferentially record treatable, rather than preventable, causes of visual impairment.

#### Incidence

No population-based cohort study was identified providing incidence data on DR in SSA. However, two cohort studies of DR in Africa were identified by this review, one of which was in SSA. A survey of diabetes complications in Mauritius was followed up 6 years later (Dowse *et al*. 1998). Of subjects with diabetes in the initial survey 40.5% were re-examined for DR (Tapp *et al*. 2006). Six-year incidence of DR was 23.8%. Duration of diabetes and fasting blood glucose were independently associated with incidence of retinopathy. Six-year progression to PDR was reported from no DR (0.4%), mild NPDR (5.2%) and moderate NPDR (29.4%).

In South Africa, a cohort of patients with insulin-dependent diabetes mellitus (IDDM) diagnosed before age 30 years was followed up over time (Gill *et al*. 1984). In those subjects seen after 10 years of follow-up, prevalence of DR had increased from 6% to 52% and PDR from 0 to 3% (Gill *et al*. 1995). In subjects seen at 20 years, prevalence of DR had increased from 12% to 59%. No incidence data were collected (Gill *et al*. 2005).

No other prospective cohort studies were identified. However, a study reflecting cumulative incidence of DR from South Africa (Distiller *et al*. 2010) reported on 1520 type 1 and 8026 t ype 2 patients who had maintained membership for ≥5 years in a community-based, privately funded diabetes management programme. In type 1 participants, the prevalence of any retinopathy at baseline and at 5 years was 22.3% and 28.0%, respectively, and in type 2 participants 20.5% and 26.6%, respectively.

### Age-related macular degeneration in Africa

#### Prevalence

The majority of data globally on AMD are from Caucasians and Asian populations (Vingerling *et al*. 1996; Cruickshanks *et al*. 2001; Buch 2005; Buch *et al*. 2005; Munoz *et al*. 2005; Arnarsson *et al*. 2006; Chen *et al*. 2008; Yasuda *et al*. 2009; Choudhury *et al*. 2011) with a paucity of data from peoples of African descent. Data on Africans are largely from studies undertaken in African populations living outside of the African continent (Leske *et al*. 2004, 2007). Comparative data between Caucasians and Africans living in the same geographical area have suggested differing predispositions towards AMD (Sommer *et al*. 1991). A single population-based study based in SSA (Kenya) determining the prevalence of AMD was identified (Mathenge *et al*. 2013). Early and late AMD prevalence in adults aged 50 years and above was 11.2% and 1.2%, respectively, amongst participants graded on digital retinal images (*n* = 3,304). After controlling for age, women had a higher prevalence of early AMD than men (odds ratio 1.5; 95% CI, 1.2–1.9), and the overall prevalence rose significantly with each decade of age (Mathenge *et al*. 2013).

#### Incidence

The incidence of AMD has been reported in population-based studies in the Americas, Australasia, Europe, and Asia; however, no data exist from the African continent to date. With the exceptions of the Latino Eye Study (Varma *et al*. 2010) and the Barbados Eye Study (Leske *et al*. 2004, 2006), all data are in Caucasian populations, and inferred data from the Barbados study suggest incident early AMD is similar to elsewhere in the world, but late AMD is less common, possibly suggesting a protective mechanism.

## Discussion

We found through our review of the literature that PSEDs are an important cause of vision loss in SSA countries. Selection bias may have led to information from French- and Portuguese-speaking countries being omitted; data from Egypt and Mauritius are unlikely to be representative for the SSA, and data not in the peer-reviewed literature were also omitted and may have been a source of bias.

The detection of and treatment for PSED poses many challenges to countries that currently lack the necessary infrastructure and resources. VISION 2020 has placed priority on conditions deemed more straightforward to treat, and this strategy has proven largely successful.

PSEDs differ from the leading anterior segment eye diseases (cataract and refractive error) in prevention/treatment, as no cures currently exist (with the exception of angle closure glaucoma). Surgical intervention can restore vision in those visually impaired from cataract, and provision of glasses can restore or improve vision in people with refractive error. However, established visual loss from PSED is difficult to reverse, and for most conditions, there is no ‘curative’ treatment.

Medical and/or surgical intervention for glaucoma can slow disease progression and thereby reduce the risk of further sight loss (Heijl *et al*. 2002). Systemic control of diabetes mellitus, retinal laser treatment, intravitreal injections and vitreoretinal surgery in sight-threatening DR can stabilise and, to some degree, improve DR status and thereby also prevent sight loss (1993). Currently no cure for AMD exists, although intravitreal therapy is available for end-stage wet AMD (approximately 10% of all AMD cases). The infrastructure required to detect AMD and deliver treatment as well as the cost of treatment itself is currently prohibitively expensive for use in most LMIC settings but is widely used in high-income countries (Bowler *et al*. 2012). Vitamin supplementation has shown some evidence of risk reduction in progression of subtypes of AMD (Evans 2006), but not prevention of AMD (Evans & Lawrenson 2012) and again may be prohibitively expensive.

This review suggests that PSEDs account for a large proportion of people with vision loss living in SSA. In recent years, improved methodologies and understanding may account for some increase in estimates of prevalence. In particular, the affordable RAAB methodology (Dineen *et al*. 2006) has led to increased numbers of researchers undertaking population-based surveys in SSA.

The majority of existing data on NCDs, including PSED, from LMIC are from cross-sectional studies providing valuable data on prevalence and risk factors. Longitudinal studies provide the opportunity to investigate the natural history of diseases, which is necessary in developing health policies at local and national levels. Few longitudinal cohort studies from LMIC have been conducted due to barriers including expense, complex logistical planning and political challenges.

A change in the focus of programme managers and policymakers over the coming decades is required if the prevalence and incidence of PSED in SSA increases as predicted. This increase is likely with extended life expectancies and success of the VISION 2020 in the treatment for anterior segment eye diseases and infectious diseases of the eye. Urbanisation and westernised lifestyles may also play a role in diseases such as diabetes and consequently DR.

Many studies worldwide have collected cross -sectional survey data on PSED prevalence; however, few studies have data on incident PSED with no SSA-based eye disease cohort studies to date. The best estimates of incidence for Africa are therefore extrapolated from studies conducted elsewhere in the world. Furthermore, investigating PSED in Africa offers a new perspective on account of the different exposures and genetic make-up of these populations compared with those studied thus far, which may reveal new insights into the cause and natural history of these diseases.

Inferring data from high-income countries undermines efforts to establish studies in LMIC, which will guide the effective use of minimal existing resources to deal with the growing burden of NCDs.

Large, community-based cross-sectional and cohort studies are needed to estimate prevalence of disease, risk factors for disease, as well as incidence and progression across Africa. Evidence for effectiveness and economics of screening of and treatment for PSED in low resource settings is vital for health service planners.
